# Resistance for Genotoxic Damage in Mesenchymal Stromal Cells Is Increased by Hypoxia but Not Generally Dependent on p53-Regulated Cell Cycle Arrest

**DOI:** 10.1371/journal.pone.0169921

**Published:** 2017-01-12

**Authors:** Jana Lützkendorf, Elisabeth Wieduwild, Katrin Nerger, Nina Lambrecht, Hans-Joachim Schmoll, Carsten Müller-Tidow, Lutz Peter Müller

**Affiliations:** Universitätsklinik und Poliklinik für Innere Medizin IV, Hämatologie/Onkologie, Martin-Luther-Universität Halle-Wittenberg, Halle (Saale), Germany; Virginia Commonwealth University, UNITED STATES

## Abstract

Adult stem cells including multipotent mesenchymal stromal cells (MSC) acquire a high amount of DNA-damage due to their prolonged lifespan. MSC may exert specific mechanisms of resistance to avoid loss of functional activity. We have previously shown that resistance of MSC is associated with an induction of p53 and proliferation arrest upon genotoxic damage. Hypoxia may also contribute to resistance in MSC due to the low oxygen tension in the niche. In this study we characterized the role of p53 and contribution of hypoxia in resistance of MSC to genotoxic damage. MSC exhibited increased resistance to cisplatin induced DNA-damage. This resistance was associated with a temporary G2/M cell cycle arrest, induction of p53- and p21-expression and reduced cyclin B / cdk1-levels upon subapoptotic damage. Resistance of MSC to cisplatin was increased at hypoxic conditions i. e. oxygen <0.5%. However, upon hypoxia the cisplatin-induced cell cycle arrest and expression of p53 and p21 were abrogated. MSC with shRNA-mediated p53 knock-down showed a reduced cell cycle arrest and increased cyclin B / cdk1 expression. However, this functional p53 knock down did not alter the resistance to cisplatin. In contrast to cisplatin, functional p53-knock-down increased the resistance of MSC to etoposide. We conclude that resistance of MSC to genotoxic damage is influenced by oxygen tension but is not generally dependent on p53. Thus, p53-dependent and p53-independent mechanisms of resistance are likely to contribute to the life-long functional activity of MSC in vivo. These findings indicate that hypoxia and different resistance pathways contribute to the phenotype that enables the prolonged lifespan of MSC.

## Introduction

During their lifelong presence the various types of adult stem cells in the human body contribute to the functional maintenance of tissues but are also exposed to a high amount of DNA-damage. Therefore, these cells are supposed to exert specific mechanisms of resistance to genotoxic damage and thereby avoid loss of functional activity as well as malignant transformation [1; 2]. Several reports indicate that p53 contributes to such resistance in gastrointestinal, hematopoietic and hair bulge stem cells [3–6].

Multipotent mesenchymal stromal cells (MSC) comprise a population of pericytic cells with adult stem cell characteristics [7]. They are present in several compartments of the human organism including bone marrow and adipose tissue [8]. Their ability of multipotent differentiation, immunomodulation and differentiation to carcinoma-associated fibroblasts (CAF) enable them to act as niche cells controlling normal tissue turnover like hematopoiesis [9]. But MSC may also contribute to malignant growth [10].

Based on their isolation mainly from bone marrow, MSC are present in the adult and elderly human body [11; 12]. Data on the impact of age on MSC frequency in vivo and their characteristics are conflicting [11; 13]. However, apart from a yet disputed role in sarcoma [14] MSC are not overtly prone to malignant transformation [15]. Therefore, MSC as other adult stem cell types need to harbor mechanisms to cope with genotoxic damage.

We and others have previously shown that MSC are resistant to genotoxic damage [12; 16; 17]. This resistance is characterized by a high threshold for apoptosis induction. Resistance is accompanied by p53 induction and proliferation arrest [12]. These results suggested a specific role for p53-regulated cell cycle arrest in MSC resistance.

Cell cycle progression and thereby resistance of cells and specifically MSC is also impacted by local oxygen tension [18; 19]. In vivo, MSC are exposed to conditions with 2 – 8% oxygen concentration [20]. Specifically, in the bone marrow MSC exist in near-hypoxic conditions [21]. We therefore hypothesized that low oxygen contributes to a p53-regulated resistance in MSC.

With the present study we aimed to characterize the role of p53 in resistance of MSC to genotoxic damage and the contribution of hypoxia to this resistance. Our data show, that resistance of MSC to genotoxic damage is increased by hypoxia but is not generally dependent on a p53-regulated cell cycle arrest.

## Material and Methods

### Cell culture and genotoxic treatment

Cultivation of MSC isolated from human bone marrow (BM) was performed as described previously [12]. All donors had given written informed consent to the additional BM aspiration according to a protocol approved by the institutional Ethics Board (Ethik-Kommision der Medizinischen Fakultät der Martin-Luther-Universität). The present study is part of the approved overall project “Untersuchung humaner adulter mesenchymaler Stammzellen in vitro und im Tiermodell (Prüfplan Vers. 2, Amendment 1 18.03.2010, Ethikvotum 20.04.2010)”. MSC growth medium was composed of low-glucose Dulbecco’s modified Eagle’s medium (DMEM) (Life Technologies, Darmstadt, Germany) with 15% fetal calf serum (FCS) and 1% penicillin/streptomycin (both PAN-Biotech, Aidenbach, Germany). Differentiation media were composed of DMEM with 10% FCS and 10 μg/ml insulin, 100 μM indomethacin, 500 μM 3-isobutyl-1-methylxanthine, 50 μM dexamethasone, 5 μM rosiglitazone for adipogenic differentiation or 200 μM ascorbic acid 2-phosphate, 50 μM dexamethasone, 10 mM glycerol-3-phosphate for osteogenic differentiation (all Sigma-Aldrich, St. Louis, USA). Growth kinetics of MSC were performed as described previously [12]. Briefly, MSC were plated with 200 cells/cm^2^ and subcultivated at 60–70% confluence. Population doublings (PD) and population doubling time (PDT) were calculated using the formulas PD = lg[(*n* cells harvested)/(*n* cells initially plated)]/lg2 and PDT = PD/(days in culture), respectively.

For genotoxic treatment MSC at passage 2 or 3 were grown to 50% confluence. The cells were overlaid with growth medium containing cisplatin (2 μM or 20 μM) or etoposide (0.75 μM; both Sigma-Aldrich). This time point was defined as day (d) 0. After 24 h the drug containing medium was removed and the cells were washed with PBS. MSC were subsequently cultivated in drug-free growth medium for later analyses (d3 or later) or harvested by trypsinization for immediate analyses (d1).

MSC cultivation under different oxygen concentrations was performed using Anaerocult® C mini for nearly physiologic (~6%, “physioxia”) and Anaerocult® A mini for hypoxic (< 0.5%) conditions according to manufacturer’s instructions (both Merck Millipore).

p53-knock down in MSC was introduced via lentiviral mediated shRNA. Lentiviral particles targeting human p53 (sc-29435-V) and control particles encoding a scrambled sequence (sc-108080) were purchased from Santa Cruz Biotechnologie. Staining for senescence-associated beta-galactosidase activity was performed according to manufacturer’s instructions using Senescence β-Galactosidase Staining Kit (Cell Signaling Technology).

The testicular germ cell tumor (TGCT) cell lines H12.1 and 2102EP were cultivated in RPMI-1640 (Sigma-Aldrich) supplemented with 10% FCS (Biochrom, Berlin, Germany) and 1% penicillin/streptomycin as described previously [12].

### Cellular platinum uptake

Cellular platinum drug accumulation was analyzed by atomic absorption spectroscopy (AAS) as described previously [22]. Briefly, MSC and TGCT cells were treated for 24 h with 3 μM cisplatin (Sigma-Aldrich). After rinsing with phosphate buffered saline (PBS; PAN-Biotech) adherent cells were harvested by trypsinization and dried. Platinum concentration was expressed as content [ppm] in lyophilized cells.

### Cell cycle analysis

Detached cells were prepared with BD CycleTEST™ PLUS DNA Reagent Kit following manufacturer’s instructions. Analyses were performed on a FACSCalibur using CellQuest software (all Becton, Dickinson and Company, San Jose, USA).

For quantification of quiescent (G0 phase) cells, DNA and RNA were differentially stained with 7-amino-actinomycin D (7-AAD) and pyronin Y [23]. Detached cells were fixed in 70% ethanol for at least 2 h at 4 °C, rinsed with PBS/1% FCS and incubated with 10 μg/ml 7-AAD (Sigma) for 20 min at room temperature. After rinsing, cells were incubated on ice for 5 min before adding 1 μg/ml pyronin Y. Following incubation for another 10 min on ice samples were acquired on a FACSCalibur.

### Flow cytometry

Flow cytometric analyses were performed as described previously [12]. The following mouse anti-human antibodies were used: Simultest™ Control y_1_/y_1_, anti-CD14-FITC (clone MΦP9), anti-CD45-PE (clone HI30), anti-HLA-DR-FITC (clone L243), anti-CD19-PE (clone 4G7), anti-CD34-FITC (clone 581), anti-CD90-PE (clone 5E10), anti-CD73-PE (clone AD2, all Becton, Dickinson and Company) and anti-CD105-FITC (clone SN6, Serotec, Oxford, UK). Analysis was performed on a FACSCalibur using CellQuest software.

### Cytotoxicity testing

Cytotoxicity was tested using the sulforhodamin-B assay [24] as described previously [12]. Briefly, MSC were seeded into 96-well plates with 1,000 cells per well in growth medium and after 24 h the cells were treated with different concentrations of cisplatin or etoposide. We implemented 2 different treatment schedules: (I) MSC were treated for 24 h, washed with PBS, incubated for 72 h with drug-free medium fixed with 10% trichloroacetic acid and (II) MSC were treated for 96 h and fixed subsequently. After staining with 0.4% sulforhodamine-B the percentage of surviving cells relative to untreated controls are represented in semilogarithmic dose-response plots.

For cytotoxicity testing under different oxygen conditions MSC were seeded in parallel into three 96-well plates with 2,000 cells per well in growth medium and the plates were brought to normoxia, physioxia and hypoxia, respectively. After 24 h the cells were overlaid with different concentrations of cisplatin and kept under the respective oxygen conditions for another 24 h and fixed subsequently.

### Comet assay

MSC were treated for 24 h with 20 μM cisplatin or etoposide. DNA strandbreaks upon cytotoxic damage were evaluated using the comet assay (alkaline single-cell gel electrophoresis) according to published protocols [25]. Short, detached cells were embedded in low melt agarose and layered on a microscope slide (10,000 cells per slide). After incubation for 1 h in lyse buffer (2.5 M NaCl, 100 mM EDTA, 10 mM TRIS, 1% triton X-100, 10% DMSO) the slides were pre-equilibrated in alkaline electrophoretic buffer (300 mM NaOH, 1 mM EDTA, pH13) for 1 h, followed by 30 min electrophorese at 25 V. Slides were rinsed in 0.4 M TRIS, pH 7.5 and stained with 10μg/ml propidium iodide.

Samples were evaluated using (I) the method of visual scoring which comprised a classification of comets according to the extent of tail DNA into class 0 (no tail) to 4 (almost all DNA in tail) [26] and (II) the description of introduced damage as% DNA in tail applying CASP Lab Software (http://casplab.com).

### Western blotting

Western blot analyses were performed as described previously [12]. The following primary antibodies were used: mouse anti-cdk1 (clone 1; 0.25 μg/ml), mouse anti-p21 (clone 70; 0.25 μg/ml), mouse anti-cyclin B (clone 18; 0.25 μg/ml, all Becton, Dickinson and Company), mouse anti-tubulin, (DM1A; 0.5 μg/ml, Dianova), mouse anti-p53 (DO-1; 0.1 μg/ml) and goat anti-actin (C-11; 0.05 μg/ml, both Santa Cruz Biotechnology, Santa Cruz, CA, USA). Immunocomplexes were visualized by enhanced chemiluminescence using horseradish peroxidase-conjugated anti-mouse IgG and anti-goat IgG (each 0.1 μg/ml, Santa Cruz Biotechnology) and Roti-Lumin (Carl Roth, Karlsruhe, Germany). Actin served as loading control.

### Statistical analysis

Statistical analysis was performed using SPSS 16.0 software (SPSS Inc., Chicago, IL, USA). If not otherwise stated depending on the equality of variances as assessed by Levene testing, results from the t-test were used in case of equal variances and results from an updated t-test were used in case of non-equal variances. In any case, a p-value < 0.05 was considered significant.

## Results

### Resistance of MSC to genotoxic damage is accompanied by a temporary cell cycle arrest and p53 target gene expression

**Fig 1:**
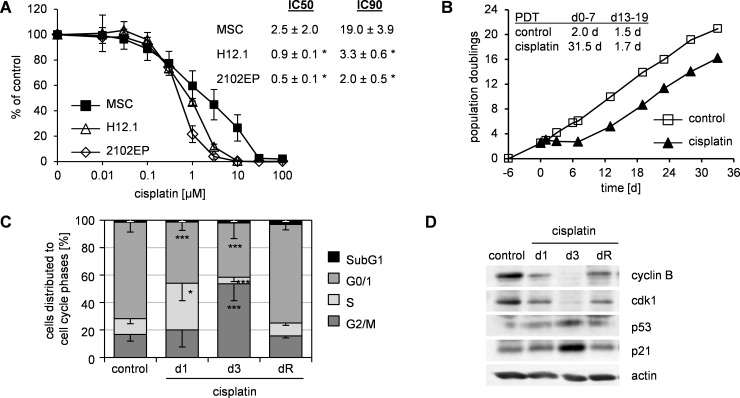
Resistance of MSC to cisplatin-induced genotoxic damage is accompanied by a cell cycle arrest. (A) SRB cytotoxicity assay for MSC and sensitive TGCT cell lines H12.1 and 2102EP. Cells were treated with cisplatin for 24 h. 72 h after end of treatment cell survival was analyzed and represented as% of untreated control in semilogarithmic dose-response plots. The respective IC50 and IC90 values are given as table insert. Mean ± standard deviation; MSC n = 9, TGCT both n = 3; * p < 0.05 vs MSC. (B) Growth kinetics of MSC after subapoptotic damage by cisplatin (24h, 2 μM on day 0). Untreated cells served as control; data are representative of 6 independent experiments; PDT–population doubling time. (C) Cell cycle analyses (propidium iodide staining) of experiments as shown in (B). On day 1, 3 and upon reconstitution of proliferation (dR) cells were harvested. Data are presented as% of cells distributed to cell cycle phases as mean—standard deviation; n ≥ 4; * p < 0.05, *** p < 0.001 vs. control. (D) Whole protein lysates from experiments as shown in (B) were analyzed by western blot. Actin served as loading control. Data are representative of 3 independent experiments.

MSC showed increased resistance to genotoxic damage in comparison to pluripotent cells. Upon cisplatin exposure MSC showed significantly higher IC50 and IC90 values than embryonic carcinoma cells TGCT H12.1 and 2102EP (Fig 1A). For subsequent mechanistic evaluations we chose a subapoptotic damage (2 to 3 μM cisplatin, 24 h).

Analysis of intracellular platinum levels revealed a rather increased cisplatin accumulation (S1A Fig) upon such damage in MSC (32.9 ± 24.7 ppm, n = 8) vs. the sensitive cell lines H12.1 (11.3 ± 0.6 ppm, n = 3; p = 0.04) and 2102EP (9.9 ± 2.3 ppm, n = 3; p = 0.03). This indicated that mechanisms independent of cisplatin uptake or efflux were involved in the resistance of MSC.

Temporary subapoptotic damage resulted in a proliferation stop but was followed by a complete restoration of proliferation (Fig 1B). At the time of restored proliferation (dR; 15.7 ± 3.6 d), cells showed typical MSC-features including phenotype and multipotent differentiation potential (S1C and S1D Fig). Analysis of cell cycle distribution revealed accumulation in S phase immediately after damage (d1) which was followed by a significant G2/M-phase accumulation on d3 and complete return to pre-treatment distribution on dR (Fig 1C, S1B Fig).

Expression analysis revealed an altered expression of p53-related cell cycle-controlling proteins with an increased expression of p53 and p21 and a reduced expression of cdk1 and cyclinB (Fig 1D). Again, a return to pre-treatment expression levels was seen on dR.

We therefore assumed that resistance to subapoptotic cisplatin-induced damage relied on a temporary, p53-dependent G2/M-cell cycle arrest.

### Hypoxia increases the resistance of MSC to genotoxic damage without impacting cell cycle

MSC are physiologically exposed to lower oxygen concentrations [20] than used for in vitro culture. Hence, we explored response to genotoxic damage at physiologic (~6%, “physioxia”) and hypoxic (< 0.5%) conditions. Under both conditions MSC maintained an unaltered immunophenotype and multipotent differentiation potential for up to 14 days (S2A and S2B Fig). In hypoxia, MSC did not proliferate but resumed proliferation after reoxygenation (S2C Fig). This proliferation stop was accompanied by a reduction in the proportion of proliferating S/G2/M phase cells and an increase in the proportion of quiescent G0 cells (S2D Fig). In contrast, physioxia did not substantially influence the proliferation of MSC.

**Fig 2:**
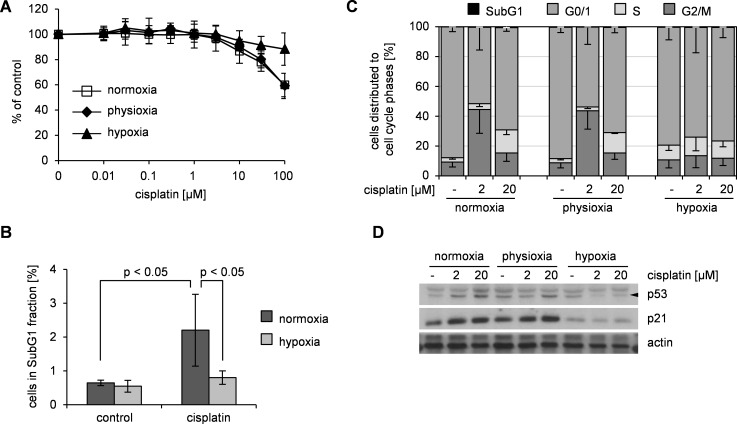
Hypoxia conferred additional resistance against cisplatin-induced damage in MSC but is not accompanied by a cell cycle arrest. (A) MSC were treated with cisplatin for 24 h under normoxic, physioxic and hypoxic conditions. Subsequently cell survival was analyzed by SRB assay and represented as% of untreated control as mean ± standard deviation in semilogarithmic dose-response plots; n = 7. (B) Normoxic and hypoxic MSC were treated 24 h with 20 μM cisplatin and fraction of apoptotic sub-G1 cells was detected by propidium iodide staining. Data are presented as% of cells in SubG1 fraction as mean ± standard deviation; n = 5. (C) MSC were treated 72 h with cisplatin under normoxic, physioxic and hypoxic conditions and analyzed for cell cycle distribution. Data are presented as% of cells in cell cycle phase as mean—standard deviation; n = 3. (D) Whole protein lysates from the experiment shown in (C) were analyzed by western blot. Data are representative of 3 independent experiments.

We determined sensitivity of MSC to subapoptotic damage under physioxic and hypoxic conditions. Analyses were performed immediately after cisplatin exposure to avoid bias of a subsequent differential proliferation at the different conditions. Upon hypoxia, MSC showed a reduced sensitivity for cisplatin compared to normoxic conditions. In contrast, physioxia did not alter the sensitivity of MSC (Fig 2A). To verify that hypoxia protects MSC, we exposed hypoxic MSC to an apoptosis-inducing damage (20 μM cisplatin, 24h). In line with a hypoxia-conferred resistance, the proportion of SubG1 cells, i. e. apoptotic cells was significantly reduced in hypoxic compared to normoxic MSC (Fig 2B).

We postulated that as for resistance upon normoxia, hypoxia-conferred additional resistance would be related to cell cycle alterations. In untreated hypoxic MSC the proportion of S-phase cells was only slightly increased compared to both, normoxic as well as physioxic MSC (Fig 2C). Upon subapoptotic damage (2μM cisplatin, 72h), the G2/M arrest as described for normoxic MSC (see Fig 1C) was similarly observed in physioxic MSC. However, hypoxic MSC showed no alterations in cell cycle distribution upon subapoptotic damage. In contrast to subapoptotic damage, apoptosis-inducing damage (20μM cisplatin, 72h) resulted in a similar cell cycle distribution in normoxic, hypoxic and physioxic MSC (Fig 2C). In accordance with these cell-cycle alterations, the expression of p21 was increased in physioxic MSC upon subapoptotic as well as the expression of p53 and p21 upon apoptosis-inducing damage similarly as in normoxic MSC. In contrast, damage of hypoxic MSC neither at subapoptotic nor at apoptosis-inducing dose resulted in an induction of p53 and p21 expression (Fig 2D).

These findings suggested that resistance to genotoxic damage in MSC at normoxic and physioxic conditions relies on p53-controlled cell cycle arrest. Thus, we hypothesized that inhibition of p53 would abrogate damage-induced arrest and increase the sensitivity of MSC.

### Knock down of p53-knock abrogates damage-induced cell cycle arrest but does not sensitize MSC

**Fig 3:**
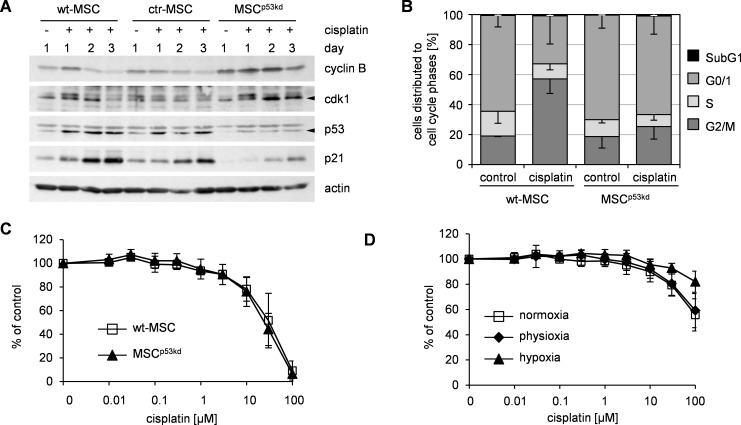
Functional p53-knock down in MSC impaired damage-induced cell cycle arrest but did neither sensitize MSC to cisplatin nor impact hypoxia-mediated resistance. (A) MSC with lentiviral p53 knock down (MSC^p53kd^), MSC with lentiviral control sh-RNA (ctr-MSC) and wildtype MSC (wt-MSC) were treated on day 0 for 24 h with 2 μM cisplatin. On the indicated days whole protein lysates were harvested and analyzed by western blot. Actin served as loading control. Data are representative of 5 independent experiments. (B) wt-MSC and MSC^p53kd^ were treated on day 0 for 24 h with 2 μM cisplatin. On day 3 cells were harvested for cell cycle analyses (propidium iodide staining). Data are presented as% of cells in cell cycle phase as mean—standard deviation; n = 3. (C) wt-MSC and MSC^p53kd^ were treated with cisplatin for 24 h. 72 h after end of treatment cell survival was analyzed by SRB assay and represented as% of untreated control as mean ± standard deviation in semilogarithmic dose-response plots; n = 3. (D) MSC^p53kd^ were treated with cisplatin for 24 h under normoxic, physioxic and hypoxic conditions. Cell survival was analyzed subsequently by SRB assay and represented as% of untreated control as mean ± standard deviation in semilogarithmic dose-response plots; n = 4.

To examine this hypothesis, we introduced a lentiviral shRNA-mediated p53-knock down in MSC (MSC^p53kd^) resulting in a sufficient reduction of protein levels (Fig 3A). In some experiments, MSC^p53kd^ showed enhanced proliferation but finally reached senescence as typical for MSC (S3A and S3B Fig). Transduction with neither sh-p53 nor control-shRNA did alter consensus MSC-characteristics (S3C and S3D Fig), however MSC transduced with control-shRNA (ctr-MSC) showed reduced proliferation in a few cases. To exclude off-target effects and given their similar proliferation (S3A Fig) wt-MSC instead of ctr-MSC were used as additional controls for further experiments.

Upon subapoptotic damage, MSC with p53 knock-down showed a reduced induction of p21 compared to controls. Also, expression of cyclin B and cdk1 was more stable in MSC^p53kd^ when compared to treated control-MSC. This expression pattern indicated that knock-down of p53 was indeed functional (Fig 3A). Again, subapoptotic damage did not alter consensus MSC characteristics in MSC^p53kd^.

Distribution of cell cycle fractions was similar in cisplatin-treated and untreated MSC^p53kd^ (Fig 3B). Therefore, in line with our hypothesis and the altered expression of regulating proteins, p53-knock-down abrogated the cell cycle arrest in response to subapoptotic genotoxic damage in MSC.

Despite this altered cell cycle response, MSC^p53kd^ and control-MSC showed identical sensitivity to genotoxic damage by cisplatin (Fig 3C), i. e. knock-down of p53 resulted in an altered cell-cycle regulation without impacting the resistance of MSC to genotoxic damage. Thus, resistance to genotoxic damage of MSC did not depend on p53-controlled cell cycle arrest.

We next wanted to test, whether the additional resistance conferred by hypoxia was also independent of p53. Sensitivity to cisplatin induced genotoxic damage was decreased in MSC with p53 knock-down upon hypoxia compared to exposure at normoxia and physioxia (Fig 3D). This paralleled the decreased sensitivity upon hypoxia as seen for wildtype MSC (Fig 2A). As for wildtype MSC no damage-induced G2/M-arrest was seen in MSC^p53kd^ upon hypoxia (S3E Fig). Accordingly, hypoxia did not result in changes of p53 and p21 expression in MSC^p53kd^ upon damage (S3F Fig).

Thus, hypoxia induced increased resistance did not depend on p53. Taken together, these data show that p53-dependent cell cycle regulation was not a general prerequisite for resistance to genotoxic damage in MSC.

### p53 knock down sensitizes MSC for damage by etoposide

Cisplatin exerts its damage through direct DNA-crosslinking. We hypothesized that p53 may impact other forms of DNA-damage in MSC. We had previously shown that MSC are resistant to etoposide [12] which induces DNA-strand breaks by topoisomerase inhibition.

Based on our previous studies we used a prolonged 96 h exposure for initial sensitivity testing in order to achieve apoptosis-inducing damage. As previously reported and as seen for cisplatin, MSC showed resistance to etoposide-related damage in comparison to sensitive pluripotent cells (S4A Fig).

**Fig 4:**
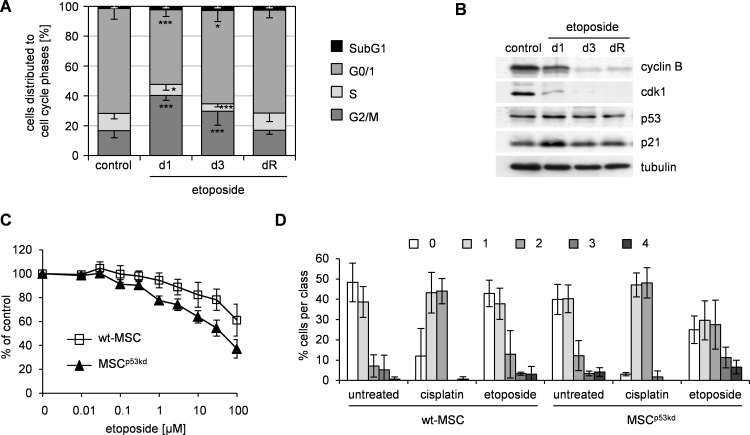
Knock down of p53 increases resistance of MSC to etoposide. (A) MSC were treated on day 0 for 24 h with 0.75 μM etoposide. On day 1, 3 and upon reconstitution of proliferation (dR) cells were harvested, respectively. Untreated cells served as control. Cell cycle analyses were performed upon propidium iodide staining. Data are presented as% of cells distributed to cell cycle phases as mean—standard deviation; n ≥ 6; * p < 0.05, *** p < 0.001 vs. control. (B) Whole protein lysates from the experiment shown in (A) were analyzed by western blot. Tubulin served as loading control. Data are representative of 3 independent experiments. (C) MSC with p53 knock down (MSC^p53kd^) and wildtype MSC (wt-MSC) were treated with etoposide for 96 h. Subsequently cell survival was analyzed by SRB assay and represented as% of untreated control as mean ± standard deviation in semilogarithmic dose-response plots; n = 3. (D) MSC^p53kd^ and wt-MSC were treated with 20 μM cisplatin or etoposide for 24 h. DNA damage was analyzed by visual scoring of comet assay (class 0 –no DNA damage, class 4 –fully damaged DNA). Respective untreated cells served as control. The diagram summarizes 3 independent experiments with a minimum of 36 analyzed comets per condition and experiment.

Similar to cisplatin a subapoptotic damage by etoposide resulted in a G2/M arrest (Fig 4A) and altered expression of cell cycle-regulating proteins with upregulation of p53 and p21 and down regulation of cyclin B and cdk1 (Fig 4B).

However, in contrast to cisplatin-conferred damage, MSC with p53-knock down showed increased sensitivity to etoposide-induced damage (Fig 4C). This resulted in significantly lower IC50 (93.3 ± 11.5 μM vs. 45.0 ± 26.0 μM, n = 3; p = 0.042) for etoposide in MSC^p53kd^ compared to control-MSC.

We hypothesized that knock down of p53 in MSC allows for more DNA-damage by etoposide but does not affect cisplatin-induced DNA-damage. To validate this, we compared DNA-double strand breaks induced by cisplatin and etoposide using the comet assay. In order to compare damage at a defined response, cells were treated at apoptosis-inducing concentrations respectively. Analysis was performed with software-based direct evaluation of tail-DNA-content (S4B Fig) as well as visual scoring (Fig 4D). Visual scoring was expressed in arbitrary units and corresponded closely to computer image-based analysis [26]. Both evaluations revealed no difference in DNA-damage between control-MSC and MSC^p53kd^ upon cisplatin treatment. In contrast, for etoposide a significant increase in DNA-damage was seen in MSC^p53kd^ compared to control-MSC (visual score: Chi^2^ analysis, p < 0.001; tail-DNA-content: t-test, p < 0.001).

Conclusively, resistance of MSC to genotoxic damage by etoposide depended on p53.

## Discussion

The main finding of our study is the observation that the resistance of MSC to genotoxic damage is enhanced by hypoxia but does not generally depend on a p53-regulated cell cycle arrest. This conclusion is derived from the following observations: i) MSC showed a relative resistance and cell cycle arrest upon cisplatin-induced damage, ii) hypoxia increased the resistance to cisplatin without inducing a cell cycle arrest, iii) p53-shRNA transduced MSC showed reduced cell cycle arrest and elevated cyclin B / cdk1 expression–thus demonstrating functional p53 knock down–but showed no altered resistance to cisplatin, iv) in contrast to cisplatin, functional p53-knock-down decreased the resistance of MSC to etoposide.

We conclude that our data reflect either the parallel activity of differential p53-dependent and p53-independent mechanisms of resistance in MSC or their activity in different subpopulations of cultivated MSC.

A resistance of MSC to different DNA-damaging agents [12; 17] as well as to radiation-induced damage [27] has been described by us and others. In accordance with our previous observation the resistance was evident at subapoptotic (i. e. IC50) as well as apoptotic (i. e. IC90) doses of DNA-damage by either cisplatin or etoposide. Our data confirm that for cisplatin this resistance is not due to reduced accumulation of damage as we observed an even higher degree of DNA-platination in MSC compared to sensitive tumor cells.

Our data show that MSC respond to genotoxic damage with an increased expression of p53 and p21 and an ensuing G2/M cell cycle arrest. A similar response has been observed in hematopoietic stem cells [28]. Thus MSC like other adult stem cells are distinct from pluripotent embryonic cells which show a p53-independent cell cycle progression [29]. The dependence on p53 is supported by the reduced expression of cdk1 and cyclin B upon genotoxic damage as p53 has been shown to inhibit the promoter activation of both genes [30].

A p53-induced cell cycle arrest may be followed by senescence as has been shown for radiation-induced damage in MSC [31]. In contrast to this, our recent and current data show that the cisplatin- or etoposide-induced cell cycle arrest in MSC is only temporary [12]. MSC resumed proliferation without alteration of their main characteristics [12].

The cisplatin-induced cell cycle arrest in MSC is p53-dependent as it was completely abrogated by knock-down of p53. This loss of damage-induced p53-dependent arrest did not affect sensitivity of MSC to cisplatin but increased their sensitivity to etoposide. We conclude, that response of MSC to genotoxic damage is not generally dependent on p53.

This is supported by the observation that hypoxia increases resistance to cisplatin-induced damage but abrogates the cisplatin-induced cell cycle arrest similarly as p53 knock down does. An effect of oxygen tension on characteristics of MSC has been well established [18; 19]. In contrast to others, we observed no effect on proliferation and resistance at physioxia, i. e. ~6% oxygen, but only at severe hypoxia. This may be explained by different culture methods. In our assays, no media change was performed, i. e. no intermittent exposure to higher oxygen concentration occurred. However, incubation at lower oxygen was much shorter than in other reports [18; 19]. Although hypoxia often is described as an inducer of p53 there are variations among different cell types [32]. Our data showed no influence of hypoxia on p53 protein level which is in accordance with other reports [19; 33]. As it was not the aim of our study no definite conclusions regarding the impact of oxygen concentration on damage resistance in MSC can be drawn. However, our data on damage at hypoxia suggested that cell-cycle arrest is not a prerequisite for resistance of MSC to cisplatin-induced damage.

Using shRNA, we achieved an efficient and functional knock down of p53 as demonstrated by protein expression for p53 and down-stream proteins p21, cyclinB and cdk1 as wells as by abrogated cell cycle arrest upon cisplatin damage. However, residual activity of p53 may have had a differential impact on response to cisplatin and etoposide as a dose dependence of p53-mediated effects has been reported [34].

Our data show that resistance to etoposide in MSC depends on p53 thereby suggesting different, damage-specific mechanisms of resistance in MSC. Which other mechanisms may contribute to this result is purely speculative. Similar to our study, p53-knock down in murine MSC was accompanied by an increased proliferation [18]. Etoposide acts as a topoisomerase II inhibitor and therefore affects only actively dividing cells whereas cisplatin-induced DNA-platination is independent of cell cycle stage. Therefore, increased proliferation upon p53 knock down may facilitate etoposide-induced damage.

A p53-independent resistance to radiation has been shown for adult hematopoietic and mammary gland stem cells and a concept of p21-directed exhaustion of damaged stem cells has been proposed [3]. Our data correspond to this concept. MSC cultivated in vitro represent an inhomogenous population [35]. Subpopulations of MSC with differential p53 activity may respond differently and less damaged MSC may compensate for damaged MSC with exhausted proliferation potential.

Taken together our data provide evidence that resistance of MSC to genotoxic damage does not generally rely on p53-dependent cell cycle regulation. Different p53-dependent and -independent mechanisms may contribute to damage-specific resistance of MSC. These data contribute to the understanding of MSC as life-long persisting niche cells with limited risk of malignant transformation.

## Supporting Information

S1 Fig Resistance of MSC to cisplatin-induced damage. (A) Platinum accumulation in MSC and TGCT cell lines upon 24h treatment with 3 μM cisplatin and analyzed by atomic absorption spectroscopy. Mean ± standard deviation; MSC n = 8, TGCT both n = 3; * p < 0.05 vs MSC. (B) Cell cycle populations from analyses as shown in Fig 1C (propidium iodide staining). Data are presented as% of cells distributed to cell cycle phases as mean ± standard deviation; n ≥ 4; * p < 0.05, *** p < 0.001 vs. control. (C) MSC after subapoptotic damage by cisplatin upon reconstitution of proliferation were analyzed for surface antigen expression by flow cytometry. Data are shown as histograms of fluorescence. Isotype controls (no filling) are overlaid on specific FITC- or PE-conjugated antibodies. Data are representative of at least 4 independent experiments. (D) MSC from (C) were incubated in growth medium (u) or specific osteogenic (o) and adipogenic (a) differentiation media. Cells were stained with alizarin pH4 and oil red for calcium deposition and lipid droplets, respectively. Data are representative of at least 4 independent experiments. Light microscopy, scale bar– 100 μm.
(TIF)

S2 Fig Effects of hypoxia on MSC characteristics. (A) MSC cultured for up to 14 days under physioxia or hypoxia were analyzed for surface antigen expression by flow cytometry. Data are representative of at least 3 independent experiments. (B) MSC from (A) were incubated in growth medium (u) or specific osteogenic (o) and adipogenic (a) differentiation media. Cells were stained with alizarin pH4 and oil red for calcium deposition and lipid droplets, respectively. Data are representative of 3 independent experiments. Light microscopy, scale bar– 100 μm. (C) Growth kinetics of MSC under normoxic, physioxic and hypoxic conditions. Cultivation under physioxia/hypoxia started on day 0. An aliquot of hypoxic cells was reoxygenated to normoxic conditions on d15. Data are representative of 5 independent experiments. (D) Cell cycle analyses of normoxic and hypoxic MSC were performed upon pyronin/7-AAD staining. Data are presented as% of cells in cell cycle phase as mean—standard deviation; n = 5.
(TIF)

S3 Fig Knock down of p53 and its effect on sensitivity of MSC to genotoxic damage. (A) Growth kinetic was performed with MSC with lentiviral p53 knock down (MSC^p53kd^), MSC with lentiviral control sh-RNA (ctr-MSC) and wildtype MSC (wt-MSC) from the same donor. Lentiviral transduction was performed on day 0. Data are representative of 4 independent experiments. (B) Late passage MSC^p53kd^ were stained for senescence-associated beta-galactosidase activity. Data are representative of 2 independent experiments. Light microscopy, scale bar– 200 μm. (C) MSC^p53kd^ were analyzed for surface antigen expression by flow cytometry. Data are shown as histograms of fluorescence. Isotype controls (no filling) are overlaid on specific FITC- or PE-conjugated antibodies. Data are representative of 4 independent experiments. (D) MSC^p53kd^ were incubated in growth medium (u) or specific osteogenic (o) and adipogenic (a) differentiation media. Cells were stained with alizarin pH4 and oil red for calcium deposition and lipid droplets, respectively. Data are representative of 4 independent experiments. Light microscopy, scale bar– 200 μm. (E) MSC^p53kd^ were treated 72 h with cisplatin under normoxic, physioxic and hypoxic conditions and analyzed for cell cycle distribution. Data are presented as% of cells in cell cycle phase as mean—standard deviation; n = 3. (F) Whole protein lysates from the experiment shown in (E) were analyzed by western blot. Data are representative of 3 independent experiments.
(TIF)

S4 Fig Resistance of MSC to etoposide-induced genotoxic damage. (A) MSC and sensitive TGCT cell lines H12.1 and 2102EP were treated with etoposide for 24 h. 72 h after end of treatment cell survival was analyzed by SRB cytotoxicity assay and is represented as% of untreated control in semilogarithmic dose-response plots. The respective IC50 and IC90 values are given as table insert. Mean ± standard deviation; MSC n = 9, TGCT both n = 6; * p < 0.05 vs MSC. (B) MSC^p53kd^ and wt-MSC were treated with 20 μM cisplatin or etoposide for 24 h. DNA damage was visualized by comet assay and calculated as tail-DNA-content using CASP Lab Software. Respective untreated cells served as control. The diagram summarizes 3 independent experiments with a minimum of 36 analyzed comets per condition and experiment. Circle—outlier; asterisk—extreme outlier.
(TIF)
