# Supplementary figures and images for: Resistance for Genotoxic Damage in Mesenchymal Stromal Cells Is Increased by Hypoxia but Not Generally Dependent on p53-Regulated Cell Cycle Arrest
Source: PLoS One. 2017 Jan 12;12(1):e0169921. doi: 10.1371/journal.pone.0169921 (PMC5231334; doi:10.1371/journal.pone.0169921)

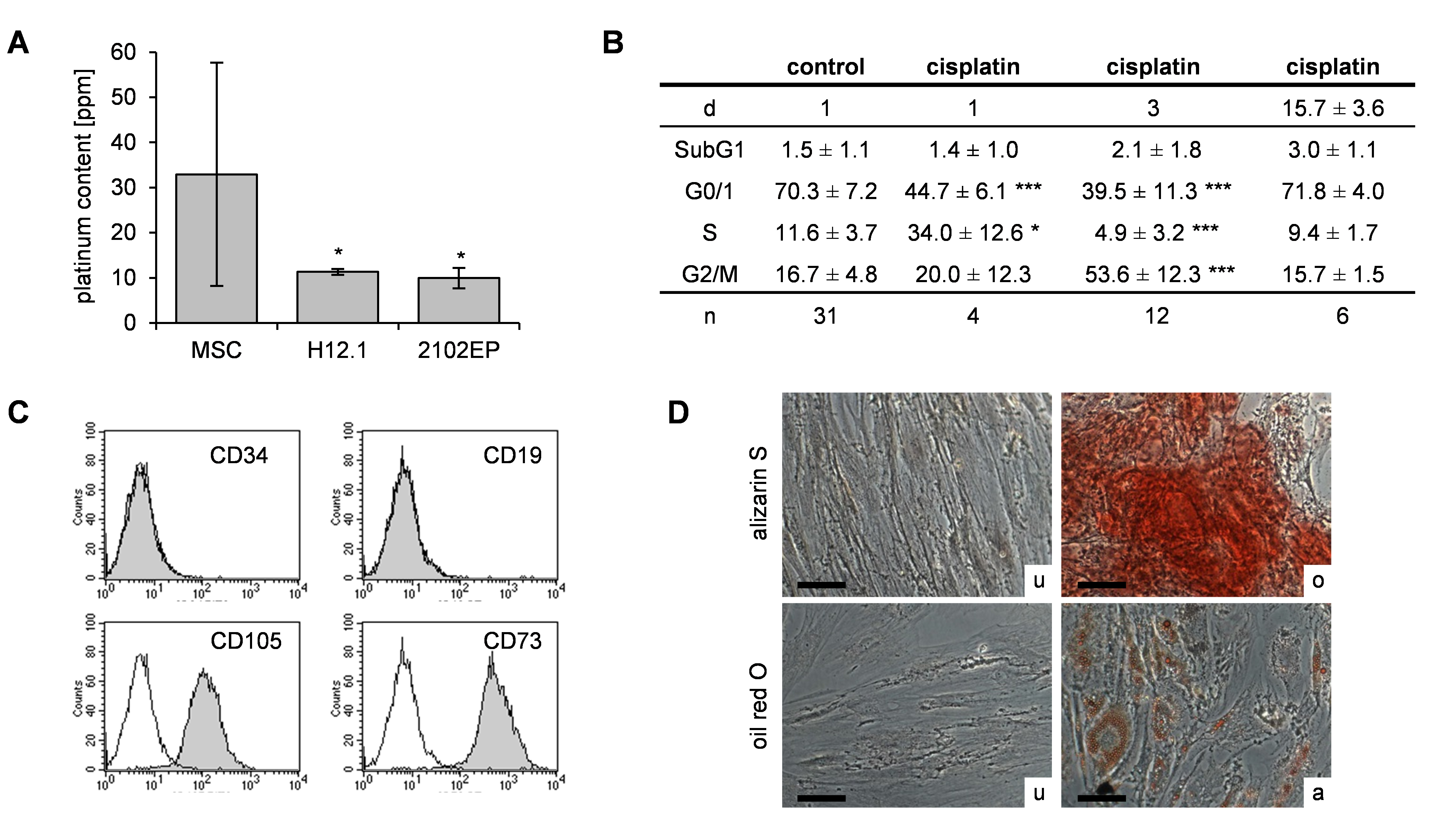

Supplement: S1 Fig — (A) Platinum accumulation in MSC and TGCT cell lines upon 24h treatment with 3 μM cisplatin and analyzed by atomic absorption spectroscopy. Mean ± standard deviation; MSC n = 8, TGCT both n = 3; * p < 0.05 vs MSC. (B) Cell cycle populations from analyses as shown in Fig 1C (propidium iodide staining). Data are presented as% of cells distributed to cell cycle phases as mean ± standard deviation; n ≥ 4; * p < 0.05, *** p < 0.001 vs. control. (C) MSC after subapoptotic damage by cisplatin upon reconstitution of proliferation were analyzed for surface antigen expression by flow cytometry. Data are shown as histograms of fluorescence. Isotype controls (no filling) are overlaid on specific FITC- or PE-conjugated antibodies. Data are representative of at least 4 independent experiments. (D) MSC from (C) were incubated in growth medium (u) or specific osteogenic (o) and adipogenic (a) differentiation media. Cells were stained with alizarin pH4 and oil red for calcium deposition and lipid droplets, respectively. Data are representative of at least 4 independent experiments. Light microscopy, scale bar– 100 μm. (TIF) [file pone.0169921.s001.tif]

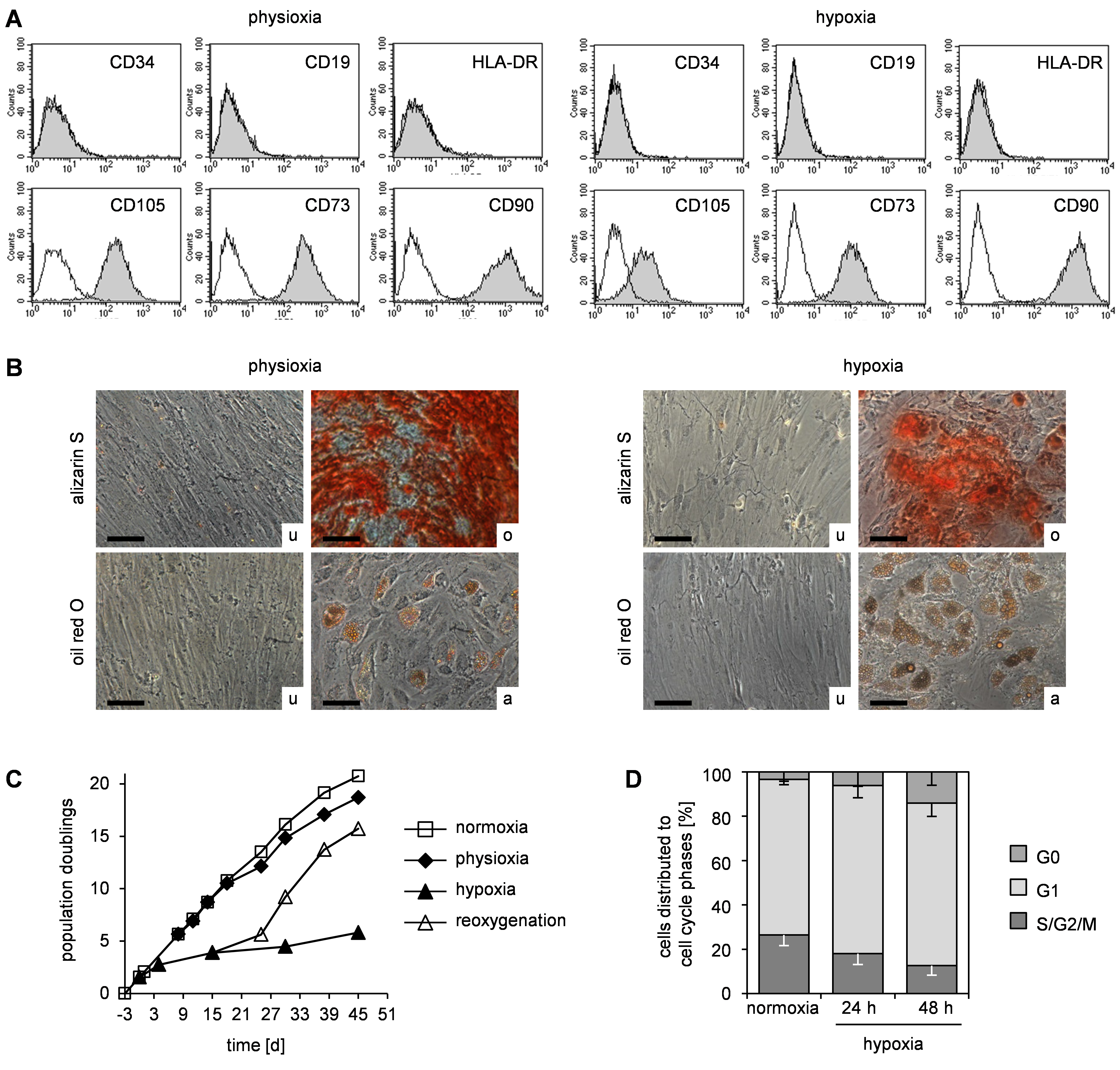

Supplement: S2 Fig — (A) MSC cultured for up to 14 days under physioxia or hypoxia were analyzed for surface antigen expression by flow cytometry. Data are representative of at least 3 independent experiments. (B) MSC from (A) were incubated in growth medium (u) or specific osteogenic (o) and adipogenic (a) differentiation media. Cells were stained with alizarin pH4 and oil red for calcium deposition and lipid droplets, respectively. Data are representative of 3 independent experiments. Light microscopy, scale bar– 100 μm. (C) Growth kinetics of MSC under normoxic, physioxic and hypoxic conditions. Cultivation under physioxia/hypoxia started on day 0. An aliquot of hypoxic cells was reoxygenated to normoxic conditions on d15. Data are representative of 5 independent experiments. (D) Cell cycle analyses of normoxic and hypoxic MSC were performed upon pyronin/7-AAD staining. Data are presented as% of cells in cell cycle phase as mean—standard deviation; n = 5. (TIF) [file pone.0169921.s002.tif]

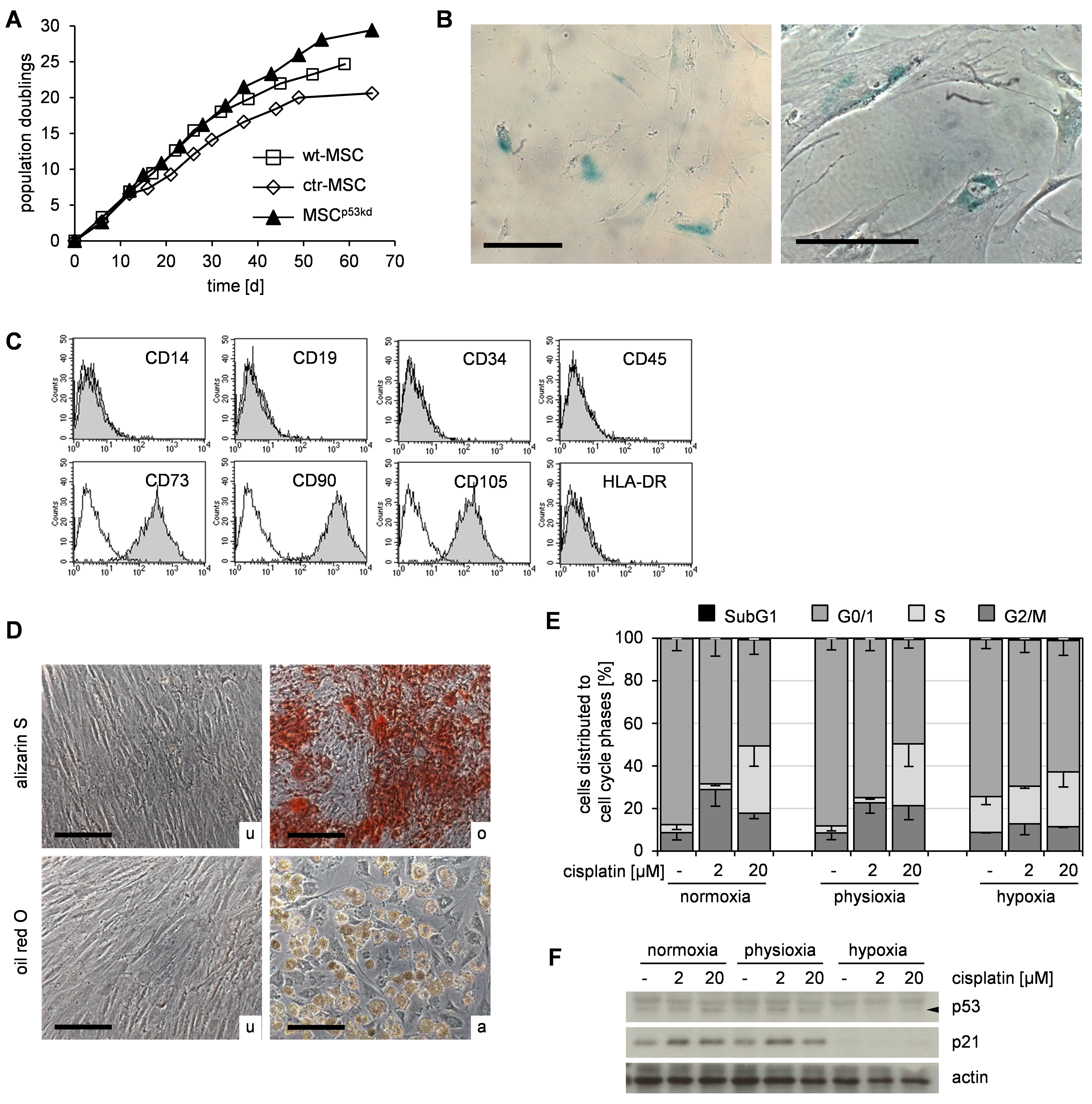

Supplement: S3 Fig — (A) Growth kinetic was performed with MSC with lentiviral p53 knock down (MSCp53kd), MSC with lentiviral control sh-RNA (ctr-MSC) and wildtype MSC (wt-MSC) from the same donor. Lentiviral transduction was performed on day 0. Data are representative of 4 independent experiments. (B) Late passage MSCp53kd were stained for senescence-associated beta-galactosidase activity. Data are representative of 2 independent experiments. Light microscopy, scale bar– 200 μm. (C) MSCp53kd were analyzed for surface antigen expression by flow cytometry. Data are shown as histograms of fluorescence. Isotype controls (no filling) are overlaid on specific FITC- or PE-conjugated antibodies. Data are representative of 4 independent experiments. (D) MSCp53kd were incubated in growth medium (u) or specific osteogenic (o) and adipogenic (a) differentiation media. Cells were stained with alizarin pH4 and oil red for calcium deposition and lipid droplets, respectively. Data are representative of 4 independent experiments. Light microscopy, scale bar– 200 μm. (E) MSCp53kd were treated 72 h with cisplatin under normoxic, physioxic and hypoxic conditions and analyzed for cell cycle distribution. Data are presented as% of cells in cell cycle phase as mean—standard deviation; n = 3. (F) Whole protein lysates from the experiment shown in (E) were analyzed by western blot. Data are representative of 3 independent experiments. (TIF) [file pone.0169921.s003.tif]

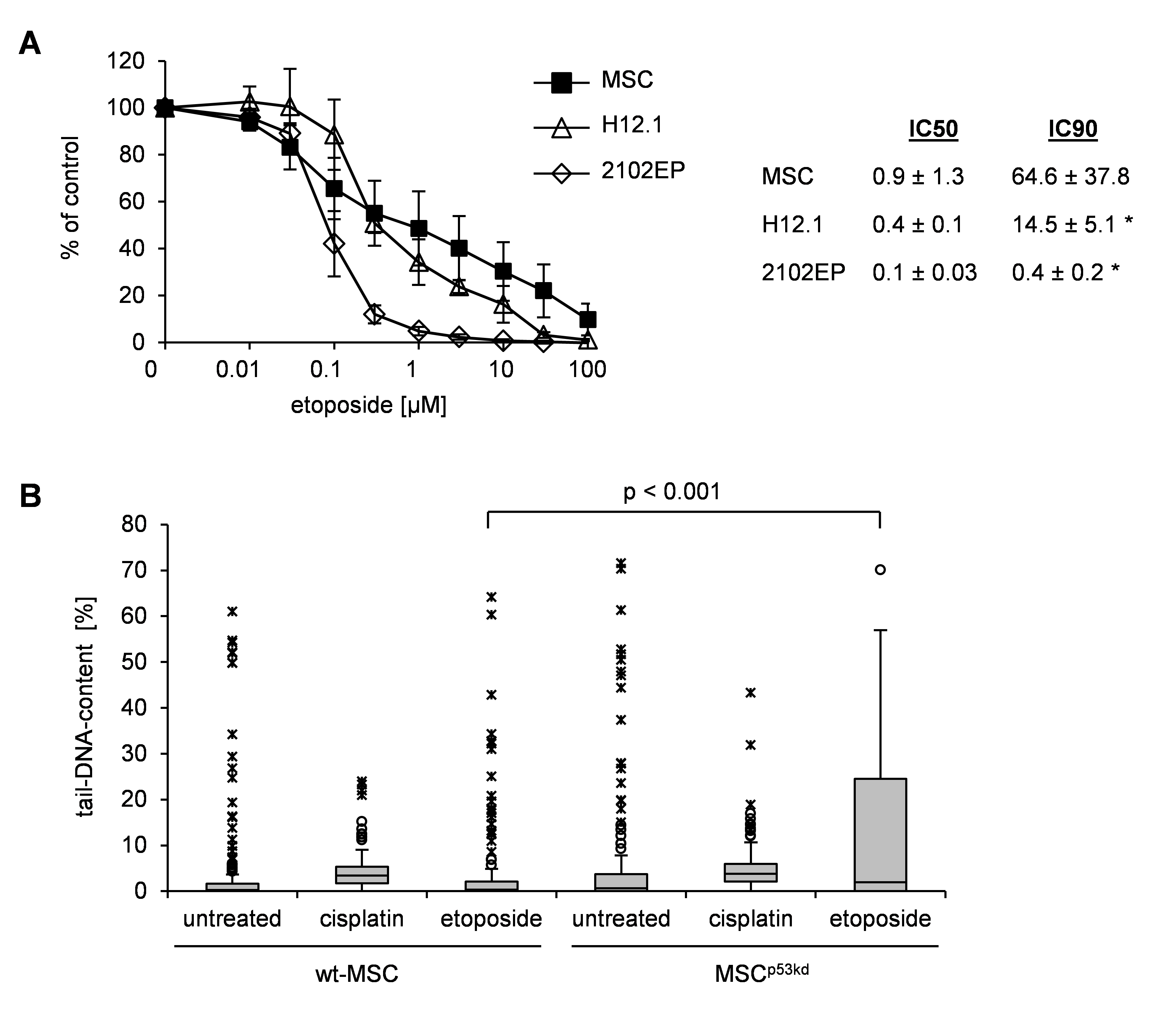

Supplement: S4 Fig — (A) MSC and sensitive TGCT cell lines H12.1 and 2102EP were treated with etoposide for 24 h. 72 h after end of treatment cell survival was analyzed by SRB cytotoxicity assay and is represented as% of untreated control in semilogarithmic dose-response plots. The respective IC50 and IC90 values are given as table insert. Mean ± standard deviation; MSC n = 9, TGCT both n = 6; * p < 0.05 vs MSC. (B) MSCp53kd and wt-MSC were treated with 20 μM cisplatin or etoposide for 24 h. DNA damage was visualized by comet assay and calculated as tail-DNA-content using CASP Lab Software. Respective untreated cells served as control. The diagram summarizes 3 independent experiments with a minimum of 36 analyzed comets per condition and experiment. Circle—outlier; asterisk—extreme outlier. (TIF) [file pone.0169921.s004.tif]
